# Targeting STAT3 enhances NDV‐induced immunogenic cell death in prostate cancer cells

**DOI:** 10.1111/jcmm.15089

**Published:** 2020-02-26

**Authors:** Xueke Wang, Xiaoyan Shao, Linaer Gu, Ke Jiang, Sitong Wang, Jianhua Chen, Juemin Fang, Xianling Guo, Min Yuan, Ji Shi, Chan Ding, Songshu Meng, Qing Xu

**Affiliations:** ^1^ Department of Medical Oncology Shanghai Tenths People's Hospital School of Medicine Tongji University Shanghai China; ^2^ Department of Radio therapy Hwa Mei Hospital University of Chinese Academy of Science Ningbo Zhejiang China; ^3^ Department of Oncology, Dermatology Hospital Tongji University Shanghai China; ^4^ Tongji University Cancer Center Shanghai China; ^5^ Institute of Cancer Stem Cell Dalian Medical University Cancer Center Dalian China; ^6^ Department of Neurosurgery Cancer Hospital of China Medical University Liaoning Cancer Hospital & Institute Shenyang China; ^7^ Department of Avian Infectious Diseases Shanghai Veterinary Research Institute Chinese Academy of Agricultural Science Shanghai China

**Keywords:** immunogenic cell death, Newcastle disease virus, prostate cancer, signal transducer and activator of transcription 3, virotherapy

## Abstract

Oncolytic Newcastle disease virus (NDV) induces immunogenic cell death (ICD), liberating danger‐associated molecular patterns (DAMPs) that provokes defiance in neoplastic malignancy. The present study aims to investigate whether and how oncolytic NDV triggers ICD in prostate cancer cells. We show that NDV/FMW, an oncolytic NDV strain FMW, elicited the expression and release of several ICD markers, that is calreticulin (CRT), heat shock proteins (HSP70/90) and high‐mobility group box 1 (HMGB1), in prostate cancer cells. Furthermore, pharmacological repression of apoptosis, necroptosis, autophagy or endoplasmic reticulum (ER) stress exerted diverse effects on the HMGB1 and HSP70/90 evacuation in NDV/FMW‐infected prostate cancer cells. Moreover, ICD markers induced in prostate cancer cells upon NDV/FMW infection, were enhanced by either treatment with a STAT3 (signal transducer and activator of transcription 3) inhibitor or shRNA‐mediated knockdown of STAT3. In nude mice bearing prostate cancer cell‐derived tumours, the tumours injected with the supernatants of NDV/FMW‐infected cells grew smaller than mock‐treated tumours. These results indicate that oncolytic NDV provokes the expression of ICD makers in prostate cancer cells. Our data also suggest that a combination of inhibition of STAT3 with oncolytic NDV could boost NDV‐based anti‐tumour effects against prostate cancer.

## 1 INTRODUCTION

Oncolytic viruses (OVs) are now being recognized as novel immunotherapeutic agents for their ability to excite antitumour immunity, in addition to triggering oncolysis.^1^, ^2^, ^3^ Multiple mechanisms are involved in OVs‐modulated cancer immunogenicity. Of note, OVs may induce immunogenic cell death (ICD), which is recently characterized with the fatality of cancer cells that provide a danger signal, that is damage‐associated molecular patterns (DAMPs) and inherent proficiencies of tumour‐relevant antigens to dendritic cells.^4^, ^5^, ^6^ Generally, the hallmarks of ICD comprise exposition of calreticulin (CRT), ATP excretion, the liberation of heat shock proteins (HSPs) and high‐mobility group box 1 (HMGB1)and induction of a type‐1 interferon response.^7^, ^8^, ^9^ Among the DAMPs, surface‐exposed CRT and exogenous ATP serve as ‘get me’ and ‘ingest me’ indications, to the immune cells, respectively.^10^, ^11^ To date, a growing number of OVs have been shown to elicit ICD in a variety of cancers.^12^, ^13^, ^14^, ^15^ We and others recently found that oncolytic Newcastle disease virus (NDV), mediates ICD in neoplastic cells of glioblastoma, lung cancer and melanoma.^16^, ^17^, ^18^, ^19^ Of translational importance, Koks et al revealed that ICD stimulation by oncolytic NDV primes adaptive antitumour immunity in a murine orthotopic immunocompetent GL261 glioma model.^16^ In addition, we recently reported that either pharmacological suppression or shRNA‐mediated knockdown of STAT3 decreased NDV‐triggered expression and release of ICD markers in melanoma cells,^19^ providing a potential mechanism to modulate NDV‐induced ICD in cancer cells. However, whether and how oncolytic NDV could induce ICD in other forms of cancer remain to be investigated.

The focus of the current investigation is to explore whether and how oncolytic NDV elicits ICD in prostate cancer cells. We present that oncolytic NDV infection provokes the main ICD markers in prostate cancer cells. Furthermore, we demonstrate that targeting signal transducer and activator of transcription 3 (STAT3) enhances the induction of ICD in prostate cancer cells upon NDV infection.

## 2 MATERIALS AND METHODS

### 2.1 Cell culture

Human embryonic kidney cells (HEK293T) were purchased from American Type Culture Collection (ATCC). Human prostate cancer cells PC‐3 and fibroblast cell line from Chicken embryo DF1 were purchased from the China Center for Type Culture Collection (Shanghai, People's Republic of China). Human prostate cancer cell line DU145 was a gift from Prof. Shujuan Shao at Dalian Medical University. HEK293T, DU145 and DF‐1 cell lines were cultured in DMEM, and PC‐3 cells were nurtured in Rosewell Park Memorial Institute (RPMI‐1640) medium, accompanied with 10% foetal bovine serum (FBS) and retained in a 37°C atmosphere containing 5% CO_2_．

### 2.2 Viral infection

An oncolytic NDV strain, NDV/FMW, was used during the study. The origin, passage and reproduction of the viruses were performed as previously described.^20^, ^21^, ^22^ The stock of NDV/FMW was quantified on DF‐1 cells and articulated as log10 50% tissue culture infective dose (TCID_50_). Prostate cancer cells having an infection at a multiplicity of infection (MOI) of 1 or 10 and recovered at several time‐points after infection.

### 2.3 Antibodies and reagents

The following antibodies were obtained from Cell Signaling Technology: HSP70, PARP, p‐STAT3 (Tyr705), HMGB1, Beclin‐1 and p‐eIF2α. Calreticulin (CRT) were bought from Abcam. Anti–haemagglutinin‐neuraminidase protein (HN) and HSP90 antibodies were purchased from Santa cruz. Goat‐anti‐rabbit antibody and anti‐β‐actin antibody were acquired from Proteintech. Goat‐anti‐mouse antibody was obtained from Bioworld. Alexa 488 and Alexa 568 were obtained from Invitrogen. Mitoxantrone, Necrostain‐1, chloroquine, Z‐VAD‐FMK, Salubrinal (Salu), C188‐9 and GSK2606414 were purchased from Selleckchem. DAPI was purchased from Beyotime. Recombinant Human interleukin‐6 (IL‐6) was purchased from PeproTech. Chloroquine (CQ) and IL‐6 were dissolved in water, and other drug stocks were solubilized in DMSO. ENLITEN^®^ATP Assay System Bioluminescence Detection Kit was obtained from Promega. HMGB1 ELISA Kit II was acquired from SHINO‐TEST CORPORATION. Cell Counting Kit‐8 was obtained from MedChem Express. Trypan Blue Solution (0.4%, K940) was purchased from Amresco.

### 2.4 Generation of lentiviral stable cell constructs

The lentiviral vectors encoding short hairpin RNAs (GIPZ‐shRNAs) targeting STAT3 and scrambled shRNA were purchased from Dharmacon (America). HEK293T cells were transfected with packaging plasmids (PSPAX and PMD2G), STAT3‐knockdown plasmid (6774) or noncoding shRNA plasmid (RHS4346) for 48 hours, the supernatants (lentiviral particles) were collected and used to directly infect DU145 and PC‐3 cells for 48 hours. Stable clones were selected using puromycin (Sigma) for 4 weeks.

### 2.5 Cell viability assay

CCK‐8 assay was used to measure the cell viability, which utilizes highly water‐soluble tetrazolium salt to produce formazan dye. 96‐well plate (8 × 10^3^ cells/well) used for cell growth followed by infection with 1 MOI NDV/FMW, while the controls were mock‐infected. Following incubation after different time periods, 10 μL of CCK‐8 solution in 100 μL of the medium was added to each well. Incubation takes place for 2‐4 hours at 37°C. After incubation, optical density (OD) was documented at 450 nm using a multiple function enzyme tabulating instrument (Enspire2300, Perkin Elmer). The OD 450 nm value was used as a measure of cell viability. The assays were repeated for three times.

### 2.6 Apoptosis

For flow cytometry analysis, cells grown in 6 cm culture dishes (1 × 10^6^ cells/dish) were infected with or without 1 MOI NDV/FMW. Cells were collected at 24 and 48 hours after infection and resuspended in 500 μL of staining solution comprising 5 μL FITC‐conjugated annexin V antibody and 5 μL PI ( 250 μg/mL stock solution). Following incubation on ice for 30 minutes, flow cytometry analysis was performed as described previously.^19^ The percentage of apoptotic cells was determined as the number of annexin V‐positive and PI‐negative cells.

For trypan blue exclusion assay, cells seeded in 12‐well plates (1 × 10^5^ cells/well) were infected with or without 1 MOI NDV/FMW for 12, 24 and 48 hours. Following trypan blue staining (0.4% trypan blue), the cells were counted under a light microscope for calculating the cell number.

### 2.7 ATP and HMGB1 assays

DU145 and PC‐3 cells were seeded in 6 cm dishes (1 × 10^6^ cells/well) with 4 mL culture medium, following NDV or mock infection, the cell‐free supernatants were collected. As per manufacturer's protocol, exogenous ATP in the supernatants was determined by using the ENLITEN ATP analysis kit (Promega). HMGB1 were detected by using the HMGB1 ELISA Kit II (Shino‐Test). The samples in ELISA assays were normalized to cell numbers.

### 2.8 Confocal Laser Microscopic Analysis

Calreticulin on the surface of the cells was detected as previously.^19^ Briefly, cells were settled and incubated with an anti‐CRT rabbit monoclonal antibody diluted 1:75 for 1 hour at room temperature, following incubation with a 1:1000 dilution of Alexa Fluor 488 goat‐anti‐rabbit IgG for 30 minutes. The nuclei were stained with DAPI (5 μg/mL). Laser scanning confocal microscope (Leica TCS SP5) was used to obtain the images. ImageJ open source was used for data evaluation on (64 Bit on Windows) imaging platform.

### 2.9 Preparation of Concentrated Supernatants

Supernatants of prostate cancer cells infected with NDV or mock‐infected were harvested, and cell junk was removed by centrifugation. Then, the supernatants (4 mL) were concentrated to 50 μL using concentrator filters (Pierce™ Protein Concentrator PES, 100K MWCO, 2‐6 mL, cat no. #88524, Thermo Fisher Scientific), according to the manufacturer's instructions.

### 2.10 Immunoblot analysis

Immunoblot (IB) analysis was performed as previously described.^19^

### 2.11 Animal experiments

BALB/c nude mice (male, 6 weeks old) were acquired from the Experimental Animal Center of Dalian Medical University and maintained in laminar flow hoods in a specific pathogen‐free environment. About 1 × 10^7^ DU145 or PC‐3 cells were subcutaneously implanted in the right flanks of mice. Measurement of tumours was done by a two‐dimensional method with the aid of Vernier calipers. The tumour volume was calculated as length × width^2^ × 0.52. Mice were separated into four groups (10 mice per group). (a) PBS control, (b) concentrated supernatants from mock‐infected cells, (c) concentrated supernatants from virus‐infected cells, which were irradiated by ultraviolet rays for 60 minutes, (d) NDV/FMW (MOI = 10). When tumours reached 100 mm^3^, the intratumoral injection was given to the tumours every third day, a total of six times. The growth rate of tumours was monitored every fifth day by digital calipers. After 40 days, mice were killed under anesthetic conditions. The animal experiments were performed in a biosafety cabinet of the SPF laboratory animal centre of the Dalian Medical University (Dalian, China). Ethical consent was approved by the Ethics Committee of Dalian Medical University.

### 2.12 Statistical Analysis

Statistical values are assumed as mean ± SD of at least three independent tests. The relevance of differences between the groups was weighed by the two‐tailed Student's *t *test. The statistical consequences were defined as *P < *.05.

## 3 RESULTS

### 3.1 Oncolytic NDV Triggers CRT exposure, HSP70/90 release as well as HMGB1 in prostate cancer cells

**Figure 1:**
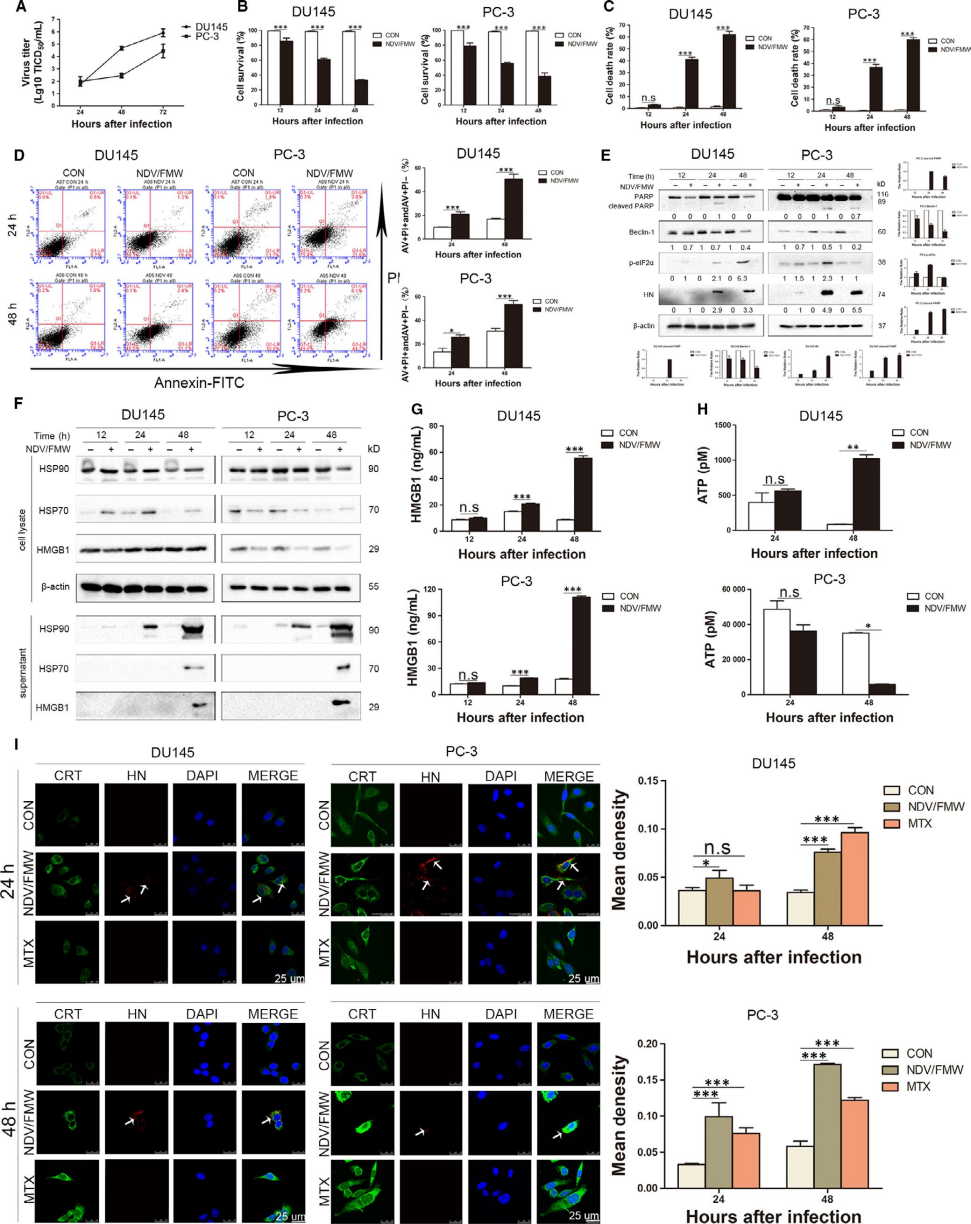
Apoptosis and immunogenic cell death were induced by NDV/FMW in prostate cancer cells. A, DU145 and PC‐3 cells were infected with 0.01 MOI NDV/FMW for 24, 48 and 72 h, viral yield was determined at the indicated times by diluting serially in DF1 cells. Values are mean ± SD from three independent tests. B, DU145 and PC‐3 cells were mock‐infected or infected with NDV/FMW (MOI = 1) for 12, 24 and 48 h. Cell viablity were assessed by the CCK‐8 assay. Values are mean ± SD. (n = 3, ***P < .001). C, Cells were treated the same as in (B). Cell death was quantified by trypan blue staining. Values are mean ± SD (n = 3, ***P < .001, n.s = not significant). D, DU145 and PC‐3 cells were mock‐infected or infected with 1 MOI NDV/FMW for 24 and 48 h. Apoptosis was determined by flow cytometry. The group of cells in Annexin V‐positive with PI‐negative quadrant and Annexin V/PI positive quadrant are illustrated. The data displayed are representative of three independent tests (*P < .05; **P < .01 and ***P < .001). E, DU145 and PC‐3 cells were treated the same as in (B). IB was used to determine the expression of the cleaved‐PARP, Beclin‐1, p‐eIF2α and haemagglutinin‐neuraminidase (HN) protein. Using β‐actin as a loading control. Protein intensity was quantified with Image Lab and shown in the histogram. All IB experiments were made three times. F, DU145 and PC‐3 cells were mock‐infected or infected with 10 MOI NDV/FMW for 12, 24 and 48 h, cell‐free supernatants (concentrated) and whole cell lysates were collected for IB analysis of HMGB1 and HSP70/90. G, DU145 and PC‐3 cells were treated the same as in (B), and cell‐free supernatants were collected to measure the amounts of HMGB1 using an enzyme‐linked immunosorbent (ELISA) detection. Existing data are mean ± SD (n = 3, n.s = not significant, ***P < .01). (H) DU145 and PC‐3 cells were treated the same as in (D), extracellular ATP in cell‐free supernatants was evaluated by ATP estimation. Existing data are mean ± SD calculated from three independent experiments (*P < 0.05; **P < .01, n.s = not significant). I, DU145 and PC‐3 cells were treated the same as in (D). The cells were assessed by immunofluorescence staining and analysed under a confocal laser‐scanning microscope. Mitoxantrine (MTX), an ICD inducer, was used as a positive control. Nucleus was stained by DAPI. NDV/FMW was detected based on HN protein. The fluorescence intensity was semi‐quantitatively determined by Image J, and the results were expressed by mean density (n = 3, *P < .05; ***P < .001, ns = not significant)

We lately described that NDV/FMW, an oncolytic NDV strain elicits ICD in lung cancer and melanoma cells.^18^, ^19^, ^23^ To analyse whether NDV/FMW infection‐induced ICD in prostate cancer cells, we initially estimated the replication of NDV/FMW and the induction of cell death in prostate cancer cell lines DU145 and PC‐3. As shown in Figure 1A, NDV/FMW robustly replicated in both cell lines. As expected, NDV/FMW infection substantially inhibited cell growth at 12, 24 and 48 hours after infection (hpi) as tested by CCK‐8 assay (Figure 1B). In addition, trypan blue staining revealed that NDV/FMW significantly triggered cell death in both two cell lines (Figure 1C). Further examination by flow cytometry showed that apoptosis was induced in both DU145 and PC‐3 cell lines upon NDV/FMW infection (Figure 1D). We further analysed the molecular markers of several forms of cell death in NDV/FMW‐infected cells. As shown in Figure 1E, pronounced cleavage of PARP, a well‐known apoptosis marker, was observed in NDV/FMW‐infected DU145 and PC‐3 cells at 24 and 48 hpi, suggesting the induction of apoptosis by NDV/FMW. However, Beclin‐1, an autophagy protein which is involved in the early stage of autophagosome formation,^24^, ^25^ was down‐regulated in NDV/FMW‐infected prostate cancer cells at 24 and 48 hpi, suggesting inhibition of autophagy (Figure 1E). In addition, NDV/FMW infection increased the phosphorylation levels of eIF2α, an endoplasmic reticulum (ER) stress marker, in DU145 cells at 48 hpi and in PC‐3 cells at 24 hpi (Figure 1E). Together, these data indicate diverse patterns of cell death involving in NDV/FMW‐facilitated growth inhibition of prostate cancer cells. We also observed the expression of NDV‐encoded haemagglutinin‐neuraminidase (HN) in both infected cell lines (Figure 1E). Taken together, these data suggest that NDV/FMW replicates and triggers cell death in prostate cancer cells.

We next determined the induction of the identified markers of ICD including the display of CRT, the release of ATP, HSP70/90 and HMGB1in prostate cancer cells upon NDV/FMW infection. As shown in Figure 1F, IB analysis of the concentrated supernatants from NDV‐infected DU145 and PC‐3 cells revealed an evident increase in the protein levels of HMGB1 and HSP70/90 at 48 hours following NDV/FMW infection compared with mock‐infected cells..

The altered HMGB1 levels were also detected by an ELISA assay (Figure 1G). Interestingly, the secretion of ATP was significantly elevated in NDV/FMW‐infected DU145 cells at 48 hpi while NDV/FMW infection of PC‐3 cells at 48 hpi significantly decreased ATP secretion (Figure 1H). In addition, confocal imaging showed a pronounced CRT expression on the cell surface of prostate cancer cells following NDV/FMW infection (Figure 1I). As expected, treatment with mitoxantrone (MTX), a known ICD inducer,^26^ increased the exposure of CRT in the tested prostate cancer cells (Figure 1I). An anti‐HN antibody was used to stain the NDV envelope protein, HN, which was apparently in virus‐infected cells but absent in uninfected or MTX‐treated cells (Figure 1I). However, although each cell was theoretically infected with NDV, in some cells the HN protein levels might be too low to be detected by immunofluorescence.

### 3.2 Cell death‐related pathways are involved in NDV/FMW‐induced release of ICD markers

**Figure 2:**
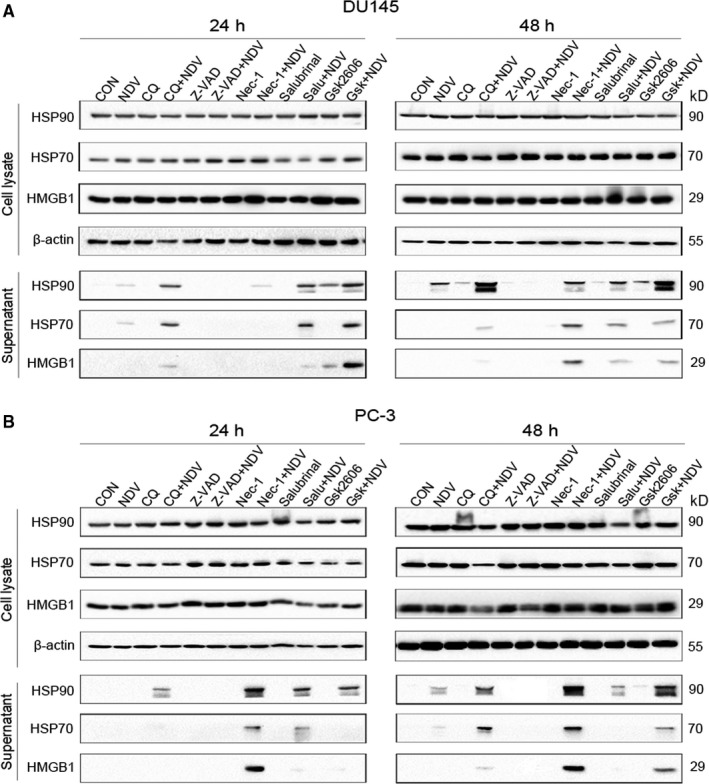
Cell death‐related signalling pathways affect the release of ICD markers upon NDV/FMW infection. A, B DU145 and PC‐3 cells were pre‐treated with various signalling pathway activators or inhibitors: autophagy inhibitor (CQ, 25 μmol/L), apoptotic inhibitor (Z‐VAD, 25 μmol/L), necrotic inhibitor (Nec‐1, 25 μmol/L), ER stress activator (Salubrinal, Salu, 15 μmol/L), ER stress inhibitor (Gsk, 1 μmol/L) or mock‐treated, later infected with NDV/FMW (MOI = 10) for 24 and 48 h. Concentrated supernatants and whole cell lysates were collected for IB analysis of HMGB1 and HSP70/90. Using β‐actin as a loading control. The illustrated data represent three independent experiments

Given the critical role of cell death in the induction of ICD markers, we explored whether pharmacological modulation of cell death would affect the induction of ICD markers in virus‐infected prostate cancer cells. To test this, we pre‐treated the cells with chloroquine (CQ, autophagy inhibitor), z‐VAD‐FMK (pan‐caspase inhibitor), Necrostain‐1 (necroptosis inhibitor) and GS42606414 (ER stress inhibitor) or Salubrinal (ER stress activator) respectively followed by NDV/FMW infection. The operative compound concentrations were chosen by a dose‐response assay for every individual compound to avoid cytotoxicity (data not shown). As shown in Figure 2A, z‐VAD‐FMK treatment attenuated the release of HMGB1 and HSP70/90 in DU145 cells infected with NDV/FMW while exposure to either CQ or Necrostain‐1 or GS42606414 boosted it. Interestingly, pretreatment with Salubrinal did not show significant effects on the release of ICD markers in NDV/FMW‐infected cells (Figure 2A). Similar results were obtained in NDV/FMW‐infected PC‐3 cells (Figure 2B).

### 3.3 Pharmacological inhibition of STAT3 augments NDV/FMW‐induced ICD markers in prostate cancer cells

**Figure 3:**
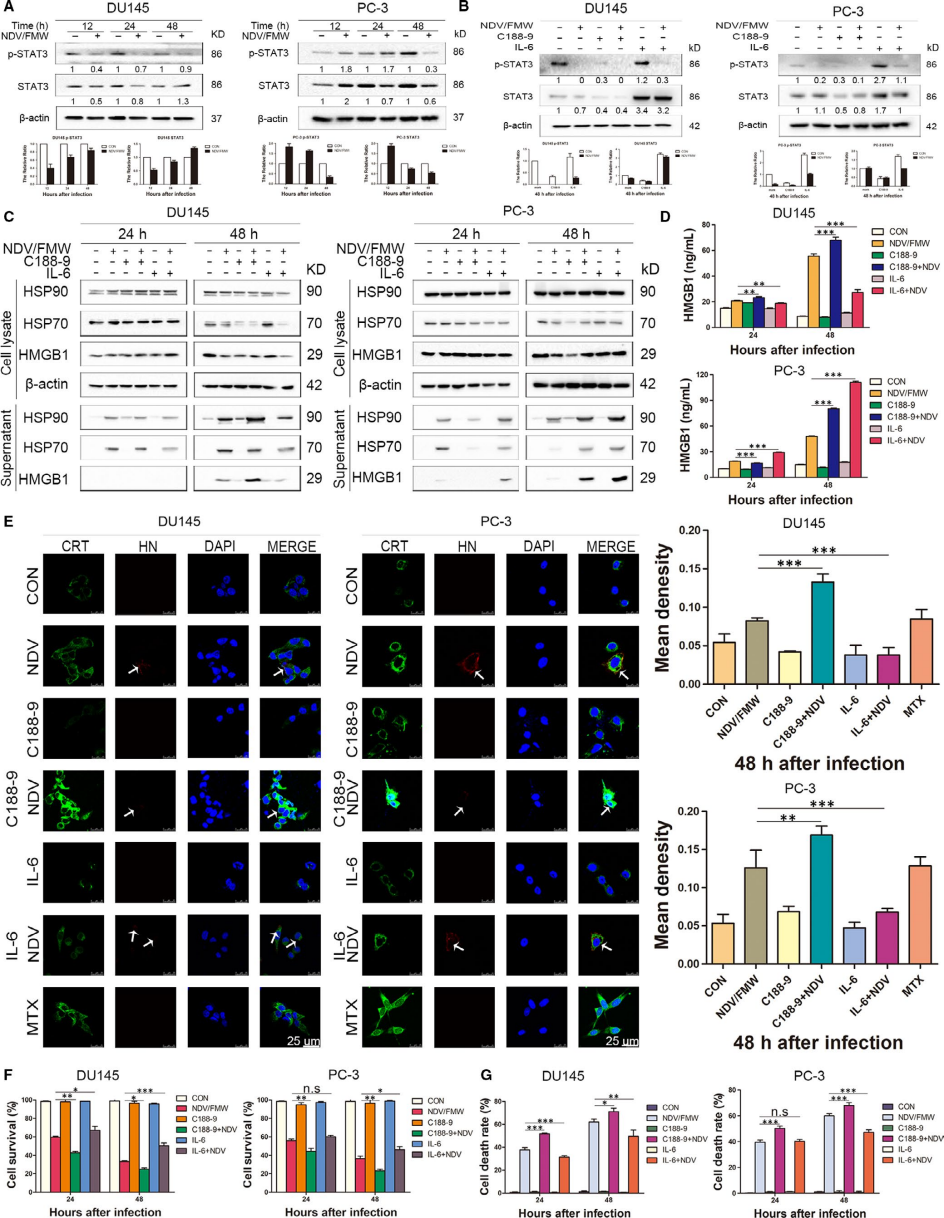
STAT3 is involved in NDV/FMW‐induced immunogenic cell death in prostate cancer cells. A, DU145 and PC‐3 cells were mock‐infected or infected with 1 MOI NDV/FMW for 12, 24 and 48 h. IB was done for the analysis of cell lysates. The relative expression of STAT3 and p‐STAT3 (Y705) was analysed by IB, and β‐actin was as a loading control. Protein intensity was quantified with Image Lab and shown in the histogram. All IB experimentations were done in triplicate. B, DU145 and PC‐3 cells were pre‐treated with STAT3 activator (IL‐6, 30 ng/mL), p‐STAT3 specific inhibitor (C188‐9, 2.5 μmol/L) or mock‐treated, following infection of 1 MOI NDV/FMW for 48 h. Cell lysates were analysed by IB for p‐STAT3 (Y705) and STAT3. Protein intensity was quantified with Image Lab and shown in the histogram. All IB experimentations were done in triplicate. C, DU145 and PC‐3 cells were pre‐treated the same as in (B), following infection with NDV/FMW (MOI = 10) for 24 and 48 h. Concentrated supernatants and whole cell lysates were collected for IB of HMGB1 and HSP70/90. D, DU145 and PC‐3 cells were pre‐treated the same as in (B), following infection with 1 MOI NDV/FMW for 24 and 48 h. The amounts of HMGB1 in the supernatants were measured using ELISA detection. Data presented are mean ± SD calculated from three independent experiments (**P < .01; ***P < .01). E, DU145 and PC‐3 cells were treated the same as in (B). All cells were assessed by immunofluorescence staining and analysed under a confocal laser‐scanning microscope. Using MTX as a positive control. Nucleus was stained by DAPI. NDV was detected based on the expression of HN protein. The fluorescence intensity was semi‐quantitatively determined by Image J, and the results were expressed by mean density (**P < .01; ***P < .001). (F) DU145 and PC‐3 cells were treated the same as in (D). The CCK‐8 assay was employed to detect cell viability. Values are mean ± SD from three independent experiments (*P < .05, **P < .01, ***P < .001, n.s = not significant). G, DU145 and PC‐3 cells were treated the same as in (D). Cell death was quantified by trypan blue staining. The experiments were done in triplicate. Values are mean ± SD (*P < .05; **P < .01; ***P < .001, n.s = not significant)

The IL‐6/JAK/STAT3 pathway is aberrantly hyperactivated in many types of cancer including prostate cancer.^27^, ^28^ Importantly, STAT3 mediates immunosuppression and inhibition of STAT3 may contribute to anticancer immunotherapy.^29^, ^30^, ^31^ Of interest, it has been shown, STAT3 deletion excites type 1 interferon response in fibrosarcoma cells, which is one of the hallmarks of ICD.^31^, ^32^ Our recent work showed that impairing STAT3 signalling impaired NDV/FMW‐induced ICD markers in melanoma cells.^19^ Thus, we hypothetically supposed that STAT3 might play a role in NDV/FMW‐induced ICD in prostate cancer cells. High STAT3 protein levels were detected in both DU145 and PC‐3 cells whereas low STAT3 levels were observed in LNCaP cells (Figure S1). To validate this, we first determined the activation of STAT3 (Y705 tyrosine phosphorylation) in virus‐infected prostate cancer cells. In Figure 3A, NDV/FMW infection resulted in a profound decrease in pSTAT3 (Y705) levels in both DU145 and PC‐3 cell lines. Next, we sought to modulate STAT3 signalling in prostate cancer cells by pharmacological inhibition. To inhibit STAT3 activity, we utilized C188‐9, a specific STAT3 inhibitor.^28^, ^33^, ^34^ As expected, C188‐9 treatment reduced the tyrosine‐phosphorylated levels of STAT3 (Y705) in both two cell lines whereas IL‐6, a known STAT3 activator, stimulated the tyrosine phosphorylation of STAT3 (Figure 3B). Figure 3C shows that pretreatment with C188‐9 profoundly augmented the release of HSP70/90 and HMGB1 in both DU145 and PC‐3 cells upon NDV/FMW infection as determined by IB. Consistently, a significant increase in HMGB1 levels was also detected by an ELISA assay (Figure 3D). Moreover, an evident increase in CRT exposure was detected in prostate cancer cells pre‐treated with C188‐9 following NDV/FMW infection (Figure 3E). Of note, C188‐9 treatment significantly enhanced NDV/FMW‐induced inhibition of growth in prostate cancer cells whereas pretreatment with IL‐6 gave the opposite effects (Figure 3F). In addition, NDV/FMW‐triggered cell death was significantly enhanced by pre‐exposure to C188‐9 (Figure 3G).

### 3.4 Knockdown of STAT3 enhances the induction of ICD markers in prostate cancer cells exposed to NDV/FMW

**Figure 4:**
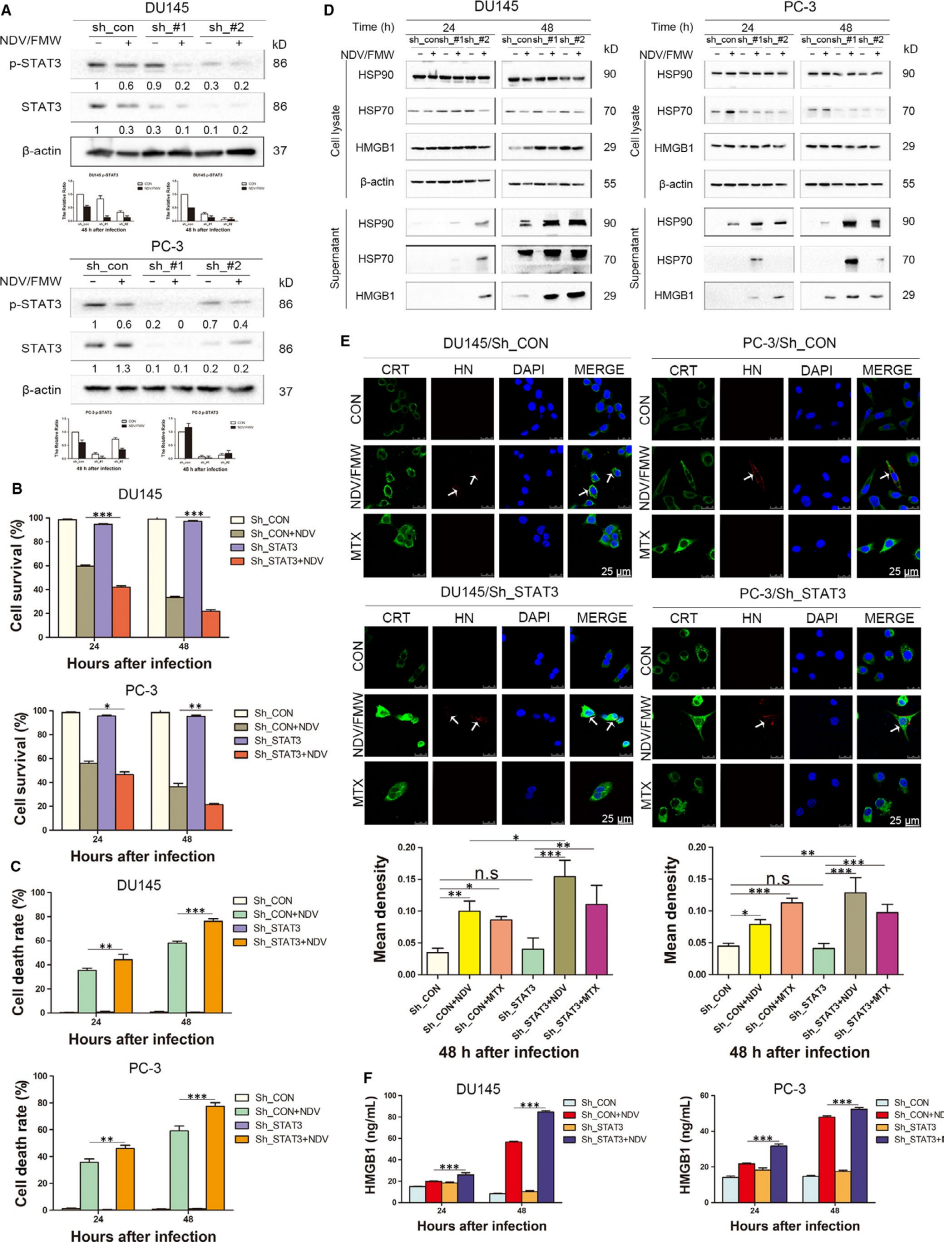
Depletion of STAT3 enhances NDV/FMW‐induced immunogenic cell death in prostate cancer cells. A, DU145 and PC‐3 cells stably knocking‐down of STAT3 were mock‐infected or infected with NDV/FMW (MOI = 1) for 48 h. The relative expression of STAT3 and p‐STAT3 (Y705) was analysed by IB, and β‐actin was used as a loading control. Protein intensity was quantified with Image Lab and shown in the histogram. All IB experimentations were made three times. B, STAT3‐depleted DU145 and PC‐3 cells were infected with or without NDV/FMW (MOI = 1) for 24 and 48 h. Cell viability was determined by the CCK‐8 assay. Values are mean ± SD (n = 3, *P < 0.05; **P < 0.01; ***P < .001). C, STAT3‐depleted DU145 and PC‐3 cells were treated the same as in (B). Cell death was quantified by trypan blue staining. The experiments were done in triplicate. Values are mean ± SD (**P < 0.01; ***P < .001). D, STAT3‐depleted DU145 and PC‐3 cells were mock‐infected or infected with 10 MOI NDV/FMW for 24 and 48 h. Concentrated supernatants and whole cell lysates were collected for IB of HMGB1 and HSP70/90. E, STAT3‐depleted DU145 and PC‐3 cells were mock‐infected or infected with NDV/FMW (MOI = 1) for 48 h. All cells were assessed by immunofluorescence staining and examined under by confocal laser‐scanning microscopy. MTX was used as a positive control. Nucleus stained by DAPI. NDV was detected based on the expression of HN protein. The experiments were done in triplicate. The fluorescence intensity was semi‐quantitatively determined by Image J, and the results were expressed by mean density (*P < 0.05; **P < .01; ***P < .001, n.s = not significant). F, STAT3‐depleted DU145 and PC‐3 cells were treated the same as in (B), and the amounts of HMGB1 in the supernatants were measured using an ELISA detection. Illustrated data are mean ± SD calculated from three independent experiments (***P < .01)

Exclusion of the possible off‐target results by C188‐9, we stably knock‐downed STAT3 in both two prostate cancer cell lines with lentiviruses‐mediated shRNAs targeting STAT3. The knockdown efficacy was verified by IB assay (Figure 4A). We observed that STAT3 knockdown significantly increased NDV/FMW‐induced inhibition of cell growth as well as cell death in infected cells compared with control cells (Figure 4B, 4). Moreover, both CRT exposure and the release of HSP70/90 and HMGB1were greatly augmented in STAT3‐depleted DU‐145 and PC‐3 cell lines upon NDV/FMW infection (Figure 4D, 4). In addition, ELISA assay revealed a significant increase in HMGB1 levels in the concentrated supernatants from NDV‐infected DU145 and PC‐3 cell lines with STAT3 depletion compare to control cell lines with STAT3 expression (Figure 4F). Of note, knockdown of STAT3 led to an increase in NDV replication in both DU145 and PC‐3 cells (Figure S2A). In addition, C188‐9 treatment increased NDV replication in DU145 cells but did not affect NDVreplication in PC‐3 cells (Figure S2B).

### 3.5 Supernatants of NDV/FMW‐infected prostate cancer cells decrease tumour growth in mice

**Figure 5:**
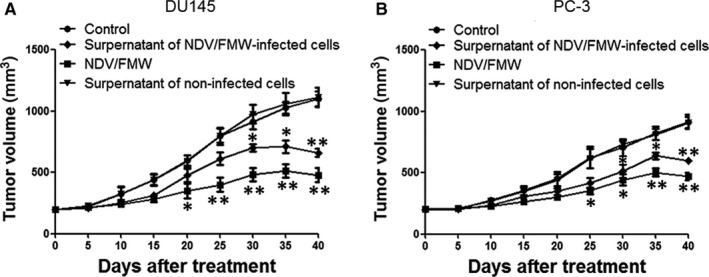
Supernatants of NDV/FMW‐infected prostate cancer cells decrease tumour growth in mice. A, B, DU145 and PC‐3 cells were introduced subcutaneously into the mice (right flanks) to establish tumours. Tumour‐bearing mice received an intratumoral injection of either PBS, the concentrated supernatants from mock‐infected cells (50 μL), the concentrated supernatants of NDV/FMW‐infected cells (50 μL) or NDV/FMW every 3 d, a total of six times. Tumour volumes were determined at 5‐d intervals for 40 d after the subcutaneous introduction and demonstrated as the Mean ± SD (n = 10) and represented as tumour volume‐time curves to show any differences in tumour regression (*P < .05; **P < .01)

Our recent work showed that the supernatants obtained from NDV/FMW‐infected melanoma cell lines repress tumour growth in vivo.^18^, ^19^ To examine whether the supernatants from NDV/FMW‐infected prostate cancer cells could inhibit prostate cancer cell growth in vivo, the supernatants of virus‐infected prostate cancer cells were harvested, concentrated and exposed to UV to deactivate the infectious virus. Tumour‐bearing mice were injected inside the tumour with the supernatants or vehicle. As illustrated in Figure 5A, the supernatants from virus‐infected cells significantly decreased the growth of DU145‐derived tumours compared with PBS‐treated or the supernatants from mock‐infected cells‐treated tumours. As predicted, NDV/FMW injection significantly repressed tumour growth (Figure 5A). Similar results were obtained in mice bearing PC‐3‐derived tumours treated as in DU145‐derived tumours (Figure 5B).

## 4 DISCUSSION

Here we provide evidence that oncolytic NDV potently induces the expression of ICD markers in prostate cancer cells in vitro. Moreover, we show that the NDV‐infected supernatants from prostate cells decreased tumour growth in a xenograft model, reinforcing that NDV‐induced prostate cancer cell death might be immunogenic. In addition, we show that targeting STAT3 augments oncolytic NDV‐elicited expression of ICD markers in prostate cancer cells. Thus, according to our knowledge, this is the first study describing the stimulation of ICD markers by oncolytic NDV in prostate cancer cells.

Currently, a variety of OVs including adenoviruses, herpes simplex virus type 1, reovirus, sendai virus, and vaccinia and fowl poxviruses have been tested in the clinical trials for the treatment of prostate cancer^15^, ^35^, ^36^ or genetically modified form (such as PSA‐cleavable rNDV), have been investigated in preclinical prostate cancer models with promising effects.^37^, ^38^, ^39^, ^40^ Our team has just described that the oncolytic NDV strain, NDV/FMW, displays cytolytic effects in a variety of cancer cells of diverse origin including lung and thyroid.^17^, ^21^, ^41^ Moreover, oncolytic NDV has been indicated to induce ICD in glioma and to elicit ICD markers in lung cancer and melanoma cells as reported by Koks *et al* and us respectively.^16^, ^18^, ^19^, ^22^ Still, whether or not oncolytic NDV elicits ICD in prostate cancer cells remains unknown. In the present study, we established that NDV/FMW infection induces the expression and release of several markers of ICD including surface‐exposed CRT, HSP70/90 and HMGB1in prostate cancer cells, indicating that NDV/FMW‐induced cytolytic effects in prostate cancer cells might be immunogenic. Thus, this study together with previous work suggests that oncolytic NDV may occupy the capacity to elicit ICD in a broad type of cancers not just limited to glioma, lung cancer and melanoma cells. It should be noted that the release pattern for ATP upon NDV infection, also the release patern for HMGB1 in the presence of IL‐6, were different between DU145 and PC‐3 cells, which might be due to the distinct genetic background of these cell lines. Given the potential of oncolytic NDV in anticancer immunotherapy as revealed by several studies,^42^, ^43^, ^44^ our study reinforces the need for further investigation of oncolytic NDV as a potent ICD inducer in immunotherapy against prostate cancer.

The activity of the transcription element STAT3 is frequently altered in prostate cancer cells. Previously, we disclosed that targeting STAT3 can inhibit tumour VEGF expression and angiogenesis in prostate cancer cells.^45^ In addition, we reported recently that STAT3 contributes to castration‐resistant prostate cancer cell survival and chemoresistance.^46^ Notably, deletion of STAT3 triggered the immunostimulatory induction of the type 1 interferon response, in fibrosarcoma cells, suggesting a key role of STAT3 in the induction of ICD.^31^, ^32^ However, whether STAT3 plays an important part in oncolytic NDV‐elicited ICD in prostate cancer cells has not been investigated. Our current data demonstrate that either shRNA‐mediated depletion of STAT3, or pharmacological inhibition of STAT3 with STAT3 inhibitor in prostate cancer cells, strikingly enhances NDV‐triggered expression and release of ICD markers, indicating that STAT3 participates with oncolytic NDV‐induced ICD in prostate cancer cells. To our surprise, the effects by STAT3 on NDV‐induced ICD markers in prostate cancer cells as presented in this study are contradictory to our recent work in melanoma cells.^19^ In that study, we found that down‐regulation of STAT3 expression or activity attenuated NDV/FMW‐induced ICD markers in melanoma cells.^19^ Therefore, the effects of STAT3 on NDV/FMW‐triggered ICD markers might be tumour origin dependent. The underlying mechanism for this diverse effect by STAT3 remains to be explored. In addition, we here found that inhibition of STAT3 enhances oncolytic NDV‐induced cell death in prostate cancer cells. Given that STAT3 inhibitors, like C188‐9, have been gauged in primary stage clinical trials for advanced‑stage cancers (NCT03195699),^28^, ^33^, ^34^ our data support that combining STAT3 inhibition with oncolytic NDV may represent a particularly promising approach to optimize NDV‐based virotherapy in the clinical setting.

## 5 CONCLUSION

Our data suggest a novel mechanism, namely induction of ICD, which could be involved in oncolytic NDV‐mediated anticancer immune response in prostate cancer. Given that oncolytic NDV‐based virotherapy overwhelms systemic tumour resistance to immune checkpoint obstruction in cancer,^47^ our study offers a rationale for the combination of oncolytic NDV with current immunotherapies, including immune checkpoint inhibitors and chemotherapeutics that induce ICD for cancer treatment.

## CONFLICT OF INTEREST

The authors confirm that there are no conflicts of interest.

## AUTHOR CONTRIBUTIONS

XKW, XYS, JS, CD, SSM and QX thought of the study, designed and performed the experiments, analysed the data and wrote the paper. LG, KJ, STW, JHC, JMF, XLG and MY performed the experiments. All authors read and approved the final manuscript.

## Data Availability

Any material and data described in this publication can be requested directly from the corresponding author, Songshu Meng.
