# Supplementary material for: Targeting STAT3 enhances NDV‐induced immunogenic cell death in prostate cancer cells
Source: J Cell Mol Med. 2020 Feb 26;24(7):4286–97. doi: 10.1111/jcmm.15089 (PMC7171322; doi:10.1111/jcmm.15089)
Supplement: Supplementary file 1 [file JCMM-24-4286-s001.docx]

Figure S1. Expression of STAT3 in prostate cancer cell lines


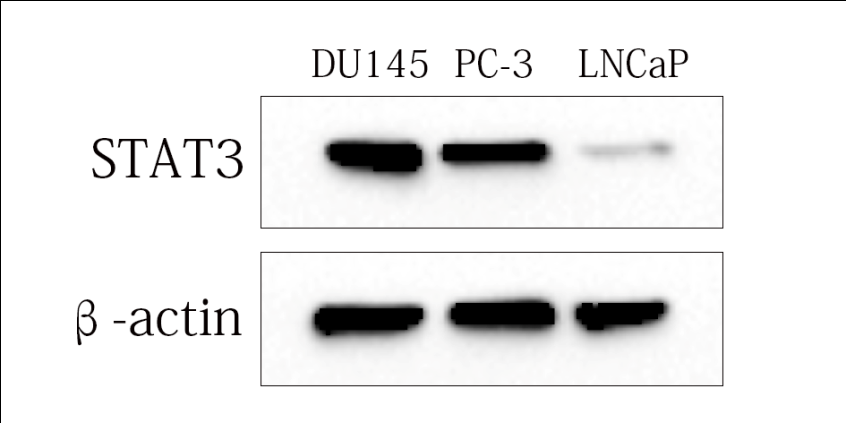

The expression of STAT3 in DU145，PC-3 and LNCaP cells was analyzed by IB, and β-actin was used as a loading control.
