# Supplementary material for: Targeting STAT3 enhances NDV‐induced immunogenic cell death in prostate cancer cells
Source: J Cell Mol Med. 2020 Feb 26;24(7):4286–97. doi: 10.1111/jcmm.15089 (PMC7171322; doi:10.1111/jcmm.15089)
Supplement: Supplementary file 2 [file JCMM-24-4286-s002.docx]

Figure S2. Effects of knock-down STAT3 or C118-9 on the replication of NDV


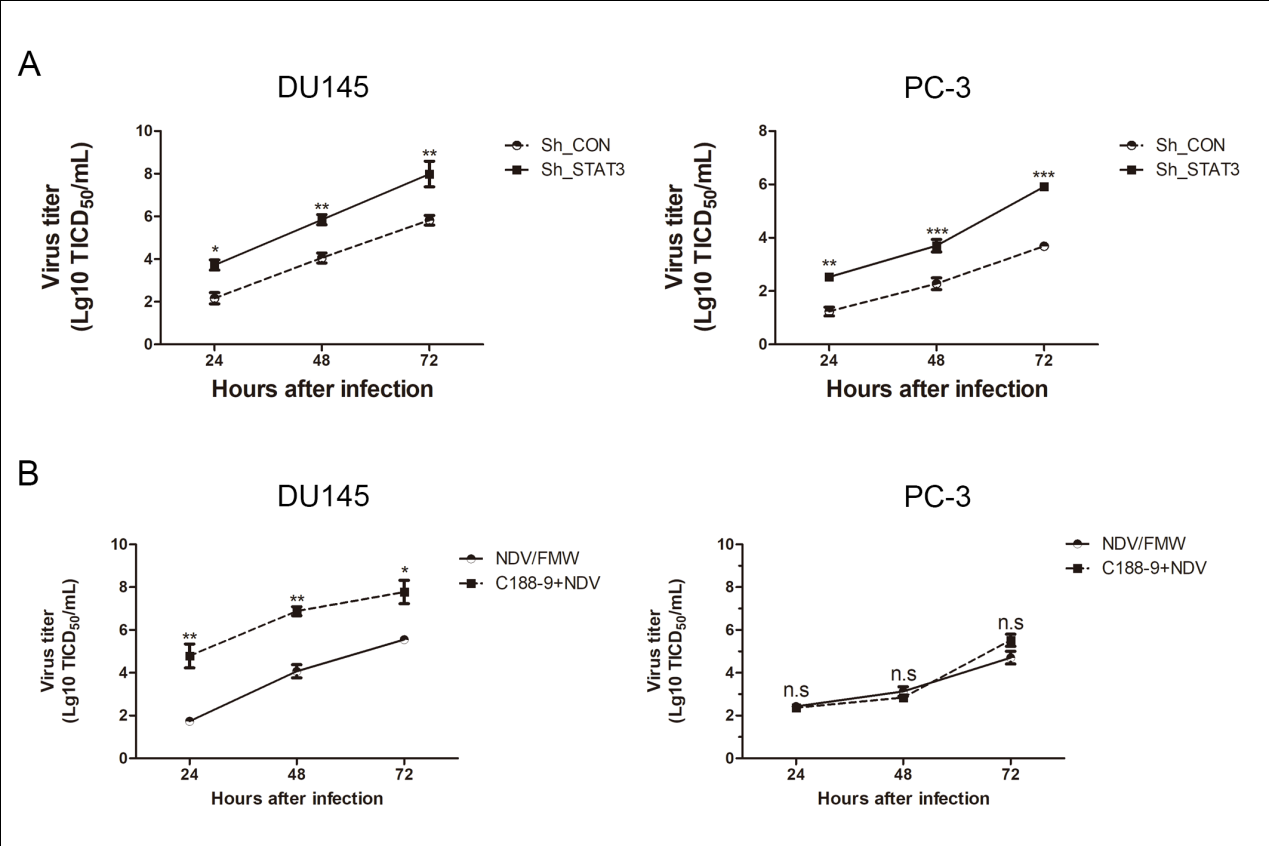


(A) DU145 and PC-3 cells stably knocking-down of STAT3 were infected with 0.01 MOI NDV/FMW for 24, 48 and 72 h, viral yield was determined at the indicated times by diluting serially in DF1 cells. Values are mean ± SD from three independent tests (*P＜0.05, **P＜0.01, ***p < 0.001) . (B) DU145 and PC-3 cells were pre-treated with p-STAT3 specific inhibitor (C188-9, 2.5 μM), following infection with 0.01 MOI NDV/FMW for 24, 48 and 72 h, viral yield was determined at the indicated times by diluting serially in DF1 cells. Illustrated data are mean ± SD calculated from three independent experiments (*P＜0.05, **P＜0.01, n.s = not significant).
